# Chain-End Reactivity and Anisotropic Electronic Softening Define Candidate Design Targets in BPDA/4,4′-ODA Polyimide Insulation

**DOI:** 10.3390/nano16191236

**Published:** 2026-09-30

**Authors:** Dongqiao Bai, Tianyu Lin

**Affiliations:** 1 Xihang University, Xi’an 710077, China; baidq@xaau.edu.cn; 2 China State Key Laboratory of Electric Insulation and Power Equipment, Xi’an Jiaotong University, Xi’an 710049, China

**Keywords:** aromatic imide oligomers, nanofilms, chain-end reactivity, reactive molecular dynamics, electric-field response, molecular orientation

## Abstract

Identifying the molecular origins of thermal and electric-field response can guide polyimide insulation design. We combined an 18-film aromatic imide oligomer ensemble, reactive molecular dynamics, and signed finite-field r^2^SCAN-3c calculations to separate packing, chain-end chemistry, and directional electronic response. Independently initialized films exhibited a fivefold variation in probe-accessible volume, with distinct connected void geometries at similar accessible fractions. Across sixteen 5 ps thermal trajectories, terminal imide N–H sites accounted for 20 of 21 persistent reference-bond losses; the nonterminal inventory contained 14 new C–O connectivities and one C–N loss. In six matched film pairs, nonterminal records increased from 2 to 5 at 1600 K and from 12 to 19 at 1800 K between 5 and 10 ps, revealing the growing contribution of nonterminal rearrangement. Trajectories extending to 20 ps also resolved the onset of increased mass escape. At ±2 V nm^−1^, the isolated ether-linked bis-imide fragment showed 0.342–0.841 eV frontier-gap narrowing across six directions, compared with at most 0.049 eV for the imide fragment, despite overlapping size-normalized induced responses. Together, these results identify chain termination and segment orientation as candidate design variables and provide quantitative descriptors for morphology-matched thermal comparisons and orientation-resolved electronic screening.

## 1. Introduction

Aromatic polyimides combine thermal resistance, mechanical integrity, and electrical insulation in power electronics, microelectronic interconnects, flexible circuits, and aerospace systems. Understanding the molecular changes that precede macroscopic deterioration is important for selecting their chemical structures and processing conditions. Breakdown strength varies with film thickness and temperature, while thermal exposure modifies charge transport and dielectric behavior [1,2,3]. Aging studies also associate chemical change with altered molecular mobility and charge transport [4]. The polyimide derived from 3,3′,4,4′-biphenyltetracarboxylic dianhydride and 4,4′-oxydianiline (BPDA/4,4′-ODA) provides a structural reference for examining how aromatic imide connectivity, molecular orientation, and chain ends contribute to these responses.

Polyimide design has traditionally treated chemical composition, packing, and polarization as connected structure–property variables. Experiments across chemically diverse polyimides have related dielectric constant and loss to polarizability, dipole content, and orientational contributions [5]. Film thickness and preferential chain alignment can change the measured dielectric response even at fixed nominal chemistry [6]. Molecular simulations provide complementary access to chain mobility, solubility parameters, and fractional free volume [7]; atomistically resolved void space [8]; and moisture-dependent chain dynamics [9]. Free-standing polyimide membranes exhibit surface density gradients [10], while short-oligomer simulations show that chemistry and water content affect packing and dynamics [11]. Recent multiscale studies have combined density functional theory (DFT), molecular dynamics (MD), and experimental measurements to improve dielectric-property correlations across broad polyimide libraries [12]. This literature suggests a useful design principle: molecular chemistry controls local electronic response, while packing and orientation determine how that response appears in a film. What remains unresolved is which structural feature first becomes chemically or electronically vulnerable in a specific nanofilm insulation system.

Chemical-site attribution is especially important for thermal simulations of short-chain models. Reactive force-field molecular dynamics (ReaxFF MD) resolves bond-order evolution [13], and C/H/O/N parameterizations have been developed for polymer carbonization [14]. ReaxFF studies have characterized product formation during accelerated polyimide pyrolysis [15] and related early bond cleavage and volatile formation to thermal stability across dianhydride and diamine combinations [16,17]. The heating rates and observation times of reactive MD differ from experimental thermogravimetric conditions [18]. Within this accelerated regime, mapping each connectivity change to its chemical site allows chain-end processes to be separated from backbone rearrangement. This distinction is particularly useful for nanofilms assembled from short molecules, where the high concentration of terminal groups can strongly influence aggregate bond-loss counts.

Electronic response provides a complementary way to resolve structural sensitivity. A local field redistributes charge and changes molecular orbital energies in a direction-dependent manner. Atomistic poling simulations have identified contributions from pendant and backbone dipoles to polyimide dielectric response [19]. Comparing molecular motifs therefore requires both size normalization and an explicit treatment of orientation. At the film scale, fluctuating-charge models such as QEq, QTPIE, and ACKS2 provide practical approximations to charge redistribution [20,21,22]. Their response direction and amplitude can be benchmarked against electronic-structure calculations, while external-field reactive simulations additionally require suitable energy removal [23]. Linking these two levels establishes a quantitative route from molecular electronic descriptors to the selection of charge models for larger-scale calculations.

Here, we connect packing variability, site-resolved thermal chemistry, and anisotropic electronic response in aromatic imide oligomer nanofilms. Eighteen independently initialized packings define the range of interface and void geometries. Zero-field reactive trajectories resolve terminal and nonterminal connectivity changes, including their evolution from 5 to 20 ps in six matched film pairs at 1600 and 1800 K. In parallel, signed finite-field calculations quantify the response of isolated imide-containing fragments and its projection onto film-normal orientations. The combined analysis identifies which structural features dominate early chemical change, how their contributions evolve with time, and which directional electronic descriptors are useful for subsequent material comparisons.

## 2. Materials and Methods

### 2.1. Nanofilm Ensemble and Nanoscale Structural Descriptors

**Figure 1:**
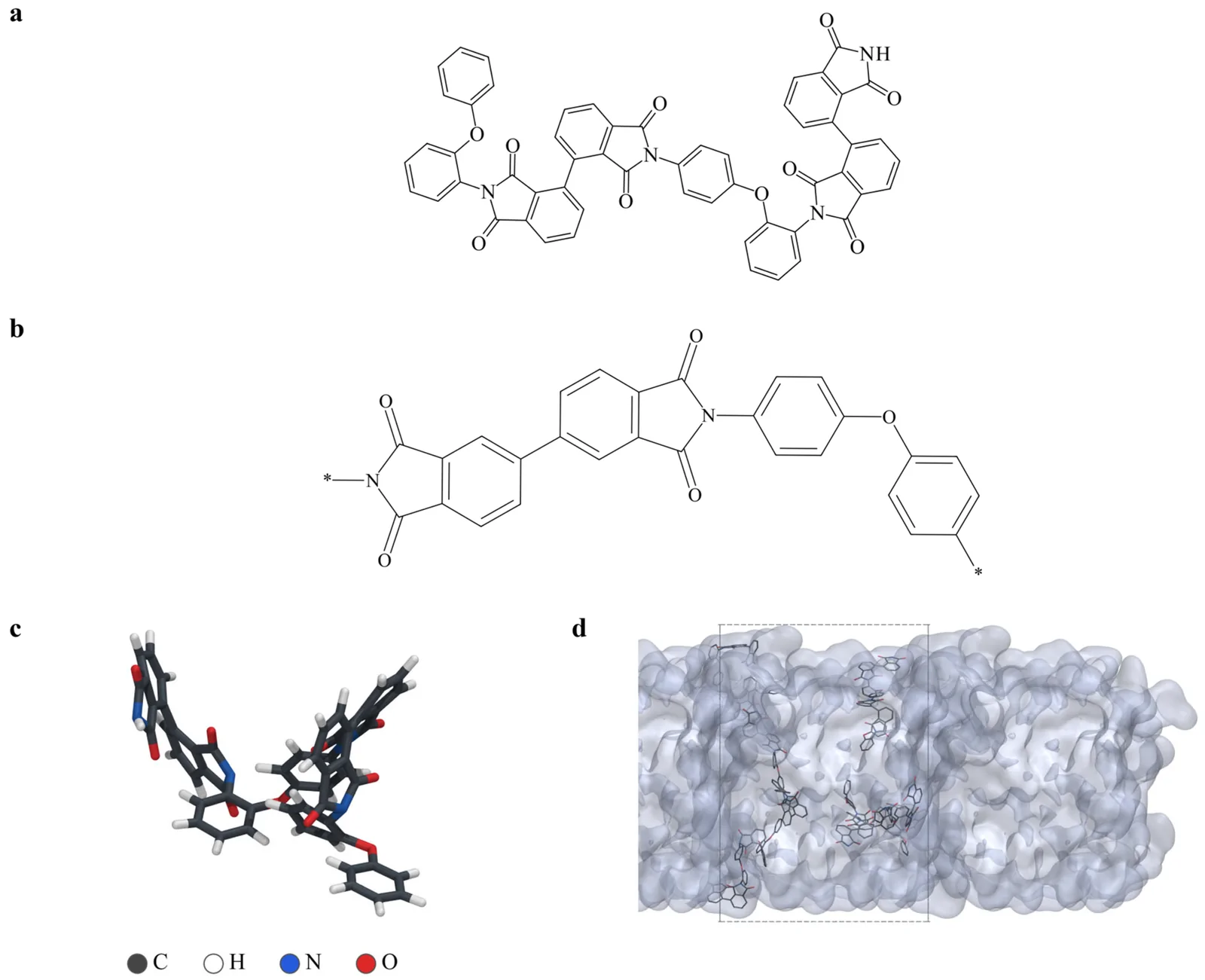
Molecular structures and nanofilm geometry. (**a**) Modeled oligomer. (**b**) BPDA/4,4′-ODA polymer repeat structure. (**c**) Oligomer conformation and element-color key. (**d**) Nanofilm side view.

Figure 1 defines the molecular model and film geometry. Each molecule was a short mixed-end aromatic imide oligomer with the Hill formula C_56_H_30_N_4_O_10_ and 100 atoms (Figure 1a). One unsubstituted terminal imide N–H was present in the C_56_H_26_N_4_O_10_ source topology, and four hydrogens were added to undercoordinated terminal aromatic carbons. These end types were tracked separately. Forty-eight molecules formed each film, resulting in 4800 atoms, 48 terminal imide N–H sites, and 5328 reference covalent edges. Each molecule contains four nitrogen atoms, one at the terminal N–H site. This end-rich short-chain architecture enables the explicit separation of terminal and nonterminal chemistry.

The input structure includes ortho N/ether substitution on some rings and imide carbonyl anchors adjacent to the biaryl linkage. Figure 1b supplies the standard BPDA/4,4′-ODA repeat structure, with para ODA and 3,3′,4,4′-BPDA connectivity, as a polymer reference; asterisks indicate chain continuation. Calculations used the oligomer in Figure 1a. Its three-dimensional conformation and the assembled film are shown in Figure 1c,d, with carbon, hydrogen, nitrogen, and oxygen represented in gray, white, blue, and red.

Eighteen films were retained from independently initialized packings (Figure 1d). Each underwent 7.1 ps of reactive preparation at 300 K, including a common final 5.0 ps locally thermostatted interval used for morphology analysis. The lateral box dimensions were 37.0 × 37.0 Å. Boundaries were periodic in x and y, while repulsive walls bounded the two free surfaces along z. Every retained film contained 48 molecular components, preserved the full reference-edge identity, and formed no new interchain covalent edge during the 300 K preparation. The prospective construction batch and all attrition are reported in Supplementary Section S1. Molecular structures and surfaces were rendered from analyzed coordinates using Visual Molecular Dynamics (VMD) 2.0 [24].

Independent initialization used separately seeded placements and orientations of the same 48 molecular templates. Orientations were sampled using normalized Gaussian quaternions, and positions were drawn within the construction region. Placements violating the element-pair minimum intermolecular separations were rejected. The resulting packings underwent common compaction and reactive preparation procedures, providing an ensemble for comparing initialization-dependent morphology at fixed molecular composition. Construction seeds, placement criteria, preparation stages, and acceptance accounting are supplied in Supplementary Section S1.

Film thickness was the separation between the mass-weighted 1st and 99th percentiles of the atomic *z* coordinates. Effective density used the film mass, lateral area, and this percentile thickness. Probe-accessible volume was evaluated between the mass-weighted 5th and 95th percentiles on a cell-centered grid with spacing no greater than 0.75 Å. Van der Waals radii were 1.70 Å for C, 1.20 Å for H, 1.52 Å for O, and 1.55 Å for N. A grid point was accessible when its distance from every atom exceeded the corresponding radius plus a 1.20 Å probe. Six-neighbor connected components were periodic laterally and nonperiodic along *z*. Surface envelopes used a lateral grid no coarser than 2.0 Å followed by a periodic 3 × 3 box average. Density, accessible fraction, largest-component fraction, component count, maximum connected film-normal span, and two-interface root-mean-square (RMS) roughness were fixed before thermal outcomes were inspected.

### 2.2. Accelerated Reactive Dynamics and Chemical-Site Attribution

Reactive trajectories used LAMMPS [25,26], the CHON-2019 C/H/O/N ReaxFF parameterization [14], and QTPIE charge equilibration. Simulations used *units real*, a 0.1 fs timestep, periodic box flags, and repulsive *wall/lepton* boundaries along *z* with ε = 1 kcal mol^−1^, σ = 3 Å, and a 6 Å cutoff. QTPIE was updated every step over 0–10 Å with a tolerance of 10^−6^ and a 200-iteration limit. Input files, force-field hashes, and software details are supplied in Supplementary Section S1.

Each branch began from its 300 K film. An all-atom NVT ramp increased the nominal bath target to 1400, 1600, or 1800 K over 0.5 ps, using a 10 fs damping constant. During the next 4.5 ps, all atoms were advanced with NVE integration, and a Langevin thermostat acted on fixed-membership surface groups, each containing 13.5% of the film mass. Energy exchange was thus imposed at the surfaces, while the core evolved without direct thermostatting. Coordinates and bond orders were saved every 0.1 ps. Core and combined-surface temperatures were calculated separately after the removal of each group’s center-of-mass motion.

The nominal temperature labels denote the surface-bath targets. During 4.5–5.0 ps, the mean core temperatures ranged across films from 1349 to 1372, 1536 to 1575, and 1725 to 1775 K at the three targets, while surface means were 1393–1412, 1595–1607, and 1796–1814 K. Each filmwise mean used 21 thermodynamic outputs. The cooler core reflects the finite-time response under surface-localized heating. Film-resolved values are reported in Table S6.

The 5 ps thermal dataset comprised sixteen completed branches from a planned 54-branch matrix: four films at 1400 K and six matched films at both 1600 and 1800 K. Four interrupted branches lacked the common 5 ps endpoint and were excluded on completion grounds; 34 branches were not started. Full run accounting is given in Supplementary Section S1. Hot-film coherence was assessed using a thickness ratio ≤ 1.20, escaped mass fraction ≤ 0.10, maximum internal z gap ≤ 2 Å, and absolute endpoint mass-center shift ≤ 2 Å, together with numerical and archive-integrity checks. The separate, stricter 300 K preparation criteria are listed in Supplementary Section S1.

A reference edge was classified as persistently lost when its bond order remained below 0.30 for at least three consecutive saved frames and the lost state persisted at the reporting endpoint. A previously absent edge was classified as persistently formed when its bond order reached at least 0.50 for three consecutive frames and the formed state persisted at that endpoint. Three frames span at least 0.2 ps. A loss and formation sharing one H atom and confirmed within 0.3 ps were grouped as a paired H relocation. This operational definition classifies connectivity and timing in the bond-order graph.

Three complementary counts were evaluated. Cumulative qualifying edge episodes count persistent reference-bond losses and new-bond formations as they arise, including episodes that recover before the endpoint. Endpoint-persistent edge records count the qualifying changes still present at the stated reporting time. Grouped pathway records combine loss and formation events involving the same H atom when their confirmation times differ by at most 0.3 ps. One paired H relocation therefore contributes two edge records and one grouped pathway record.

Every event atom was mapped to the 100-atom input topology by element-labeled graph isomorphism. Records were partitioned into terminal imide N–H and nonterminal classes. C–O formations were assigned to imide carbonyl O, imide carbonyl C, biphenyl aromatic C, or ether-linked aromatic C, and their shortest graph distance from terminal imide N was recorded. Event-definition sensitivity was evaluated by reanalyzing the same trajectories with formation thresholds of 0.40 and 0.60 and persistence requirements of two and five frames.

Time dependence was evaluated for all six matched films at 1600 and 1800 K through a total elapsed time of 20 ps, with scheduled endpoints at 5, 10, 15, and 20 ps. The 5–20 ps interval began from the corresponding 5 ps checkpoints and retained the molecular connectivity, CHON-2019 force field, QTPIE settings, 0.1 fs timestep, repulsive walls, and fixed surface groups. Coordinates and velocities were continuous at the checkpoint interface. The restart files did not preserve the Langevin random-number state, so deterministic thermostat seeds were specified before continuation. These are checkpoint-based stochastic continuations.

The same bond-order, persistence, and shared-H grouping criteria were applied at every reporting time. Persistence was evaluated separately at each endpoint, so an earlier change could disappear from a later inventory if its connectivity recovered. All scheduled trajectories were included. Numerical completion and hot-film coherence were evaluated separately; endpoints following the first coherence-threshold crossing were retained as post-coherence-loss diagnostics in the filmwise tables.

### 2.3. Signed Finite-Field Response, Orientation Projection, and Statistics

Neutral singlet imide-containing (C_14_H_9_NO_2_, 26 atoms), biphenyl bis-imide (BI; C_16_H_8_N_2_O_4_, 30 atoms), and ether-linked bis-imide (EI; C_28_H_16_N_2_O_5_, 51 atoms) fragments were extracted from the input oligomer geometry and hydrogen-capped. Fixed-nucleus r^2^SCAN-3c single point calculations [27] were performed with ORCA 6.1.0 [28]. The primary matrix contained zero field and ±2 V nm^−1^ along molecular-coordinate x, y, and z. Inputs used TightSCF, SlowConv, 300 self-consistent-field (SCF) iterations, a center-of-mass field origin, Hirshfeld charges [29], and Mayer bond orders. The field conversion was 514.220674763 V nm^−1^ per atomic unit, so 2 V nm^−1^ corresponded to 0.003889380762 atomic units.

For a field applied along coordinate *j*, the signed induced-dipole vector was [**μ**(+E*j*) − **μ**(−E*j*)]/2. The three vectors formed the columns of a molecular induced-response matrix. Its negligible antisymmetric residual allowed for the use of the symmetric part and its principal values. Responses were divided by the number of parent heavy atoms retained in each fragment to reduce simple size bias. The axial frontier-gap response was separated into field-even narrowing, *g*(0) − [*g*(+E) + *g*(−E)]/2, and the field-odd shift, [*g*(+E) − *g*(−E)]/2. Signed off-axis pairs along (*x* + *y*)/√2, (*x* + *z*)/√2, and (*y* + *z*)/√2 were added to test a quadratic directional representation of the even gap response.

Fragment topology was mapped to each molecule in the 18-film ensemble. A Kabsch fit of parent heavy atoms to the 300 K coordinates supplied the rigid-reference orientation. Rotating the induced-response tensor into the laboratory frame yielded its film-normal projection. Fit residuals quantified conformational differences between the reference fragments and their placements in the films.

QEq, QTPIE, and ACKS2 were evaluated at the same fragment geometries in nonperiodic zero-step LAMMPS calculations at ±2 V nm^−1^. Their signed induced dipoles and atomwise charge ordering were compared with r^2^SCAN-3c to benchmark immediate electronic redistribution.

The film was the independent statistical unit. Distributions were summarized using individual values, medians, interquartile ranges, and observed ranges. Matched 1600–1800 K contrasts used two-sided exact sign-flip tests. The six paired films remained the independent units across reporting times. Given this small completed subset, temperature contrasts were interpreted descriptively. Four prespecified morphology descriptors were screened against total event burden at each temperature using exact Spearman permutation tests. The post hoc end-group-excluded screen comprised eight tests, assessed by Benjamini–Hochberg adjustment and leave-one-film-out checks. Detailed statistical and sampling information is provided in Supplementary Sections S1 and S2.

## 3. Results and Discussion

### 3.1. Packing Heterogeneity and Connected Void Space

**Figure 2:**
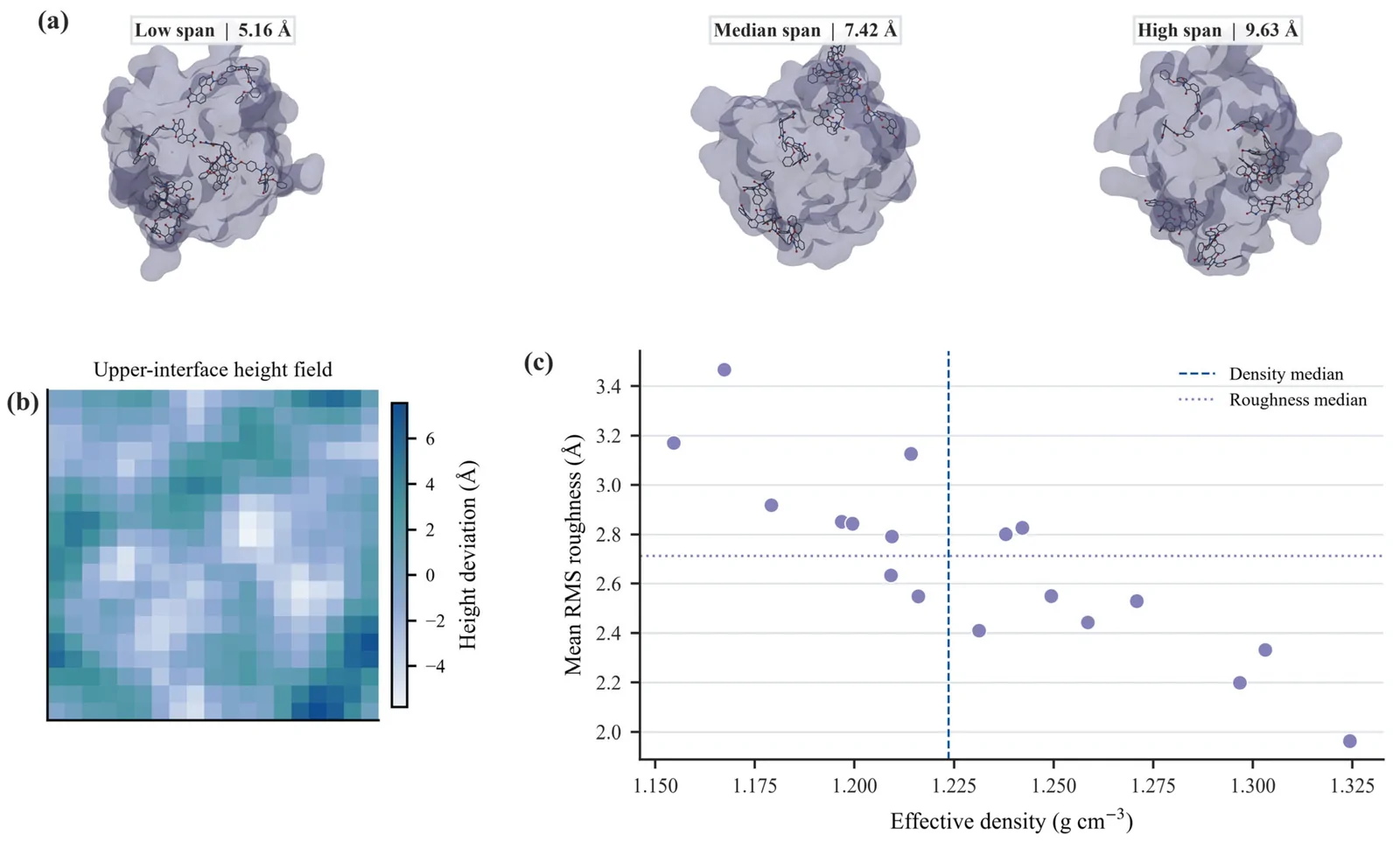
Packing and interface heterogeneity. (**a**) Span-selected nanofilm renderings. (**b**) Upper-interface height field. (**c**) Filmwise density and roughness.

Independent initialization produced distinct film morphologies at fixed composition. Effective density ranged from 1.155 to 1.324 g cm^−3^, with a median of 1.224 g cm^−3^, while two-interface RMS roughness ranged from 1.96 to 3.47 Å. A simulated bulk BPDA/4,4′-ODA density of 1.297 g cm^−3^ [30] provides contextual comparison across different molecular architectures and boundary conditions. Figure 2a illustrates films near the lower, median, and upper values of the connected film-normal void span. Their different surface shapes are resolved by the upper-interface height field in Figure 2b and the ensemble density–roughness distribution in Figure 2c, where reference lines indicate medians.

**Figure 3:**
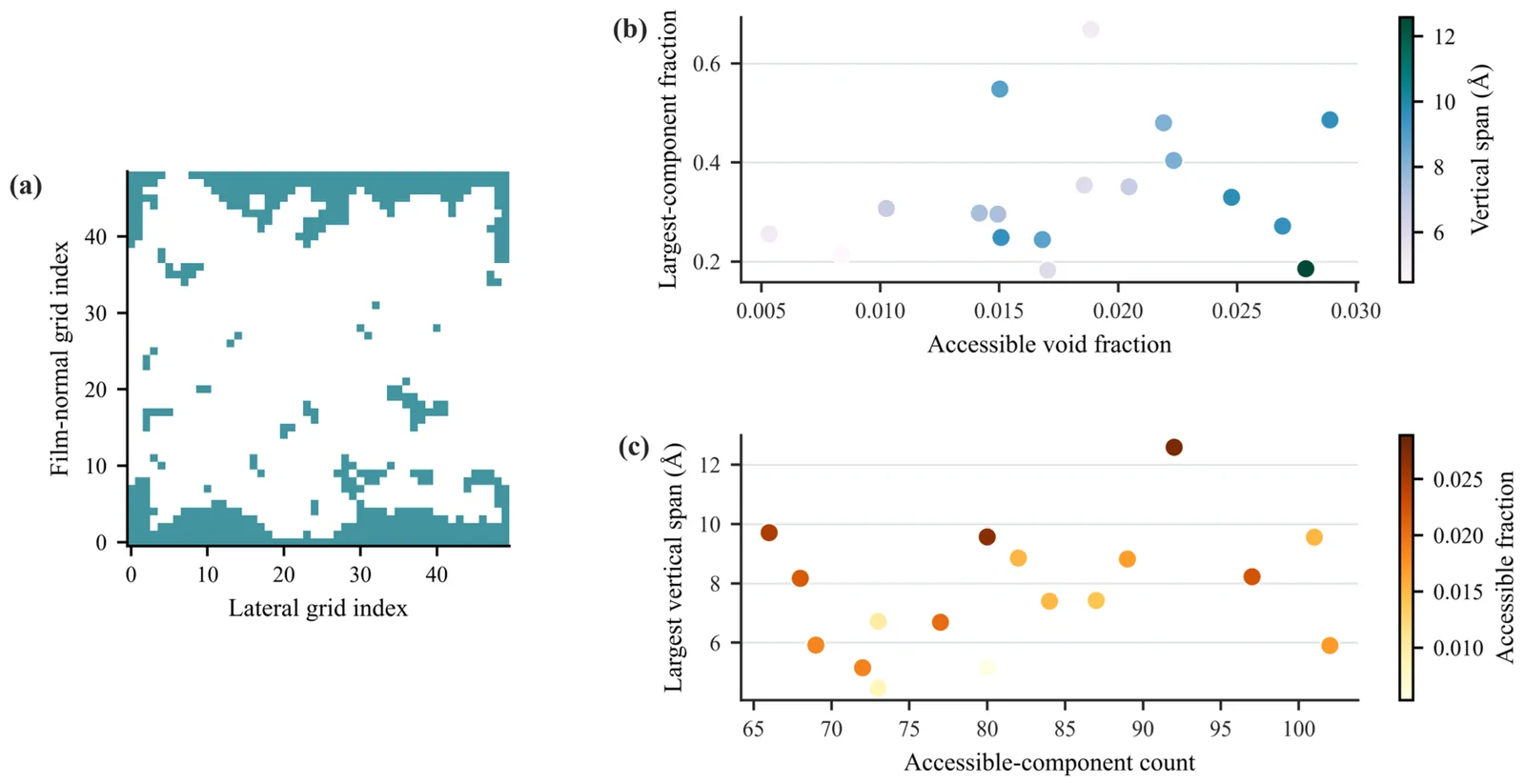
Probe-accessible volume topology. (**a**) Accessible grid projection. (**b**) Volume-connectivity relation. (**c**) Component count and film-normal span.

Void descriptors varied even more strongly. The probe-accessible fraction ranged from 0.0053 to 0.0289, a more than fivefold difference. The largest component contained 0.183–0.669 of all accessible grid points, and the maximum connected film-normal span ranged from 4.47 to 12.59 Å. Figure 3a shows the film-normal projection of the accessible grid for a representative structure. The total accessible fraction did not uniquely determine component dominance (Figure 3b), and component count did not uniquely determine film-normal continuity (Figure 3c). Films with similar accessible fractions therefore had different connected void geometries.

The accessible fraction and connected span therefore capture complementary aspects of film packing: how much void space is available and how that space extends through the film. Retaining both descriptors distinguishes packings that would appear similar under a single free-volume measure. This provides a practical basis for morphology-matched comparisons of chemically identical nanofilms.

### 3.2. Chain-End Attribution and Time-Dependent Connectivity

**Figure 4:**
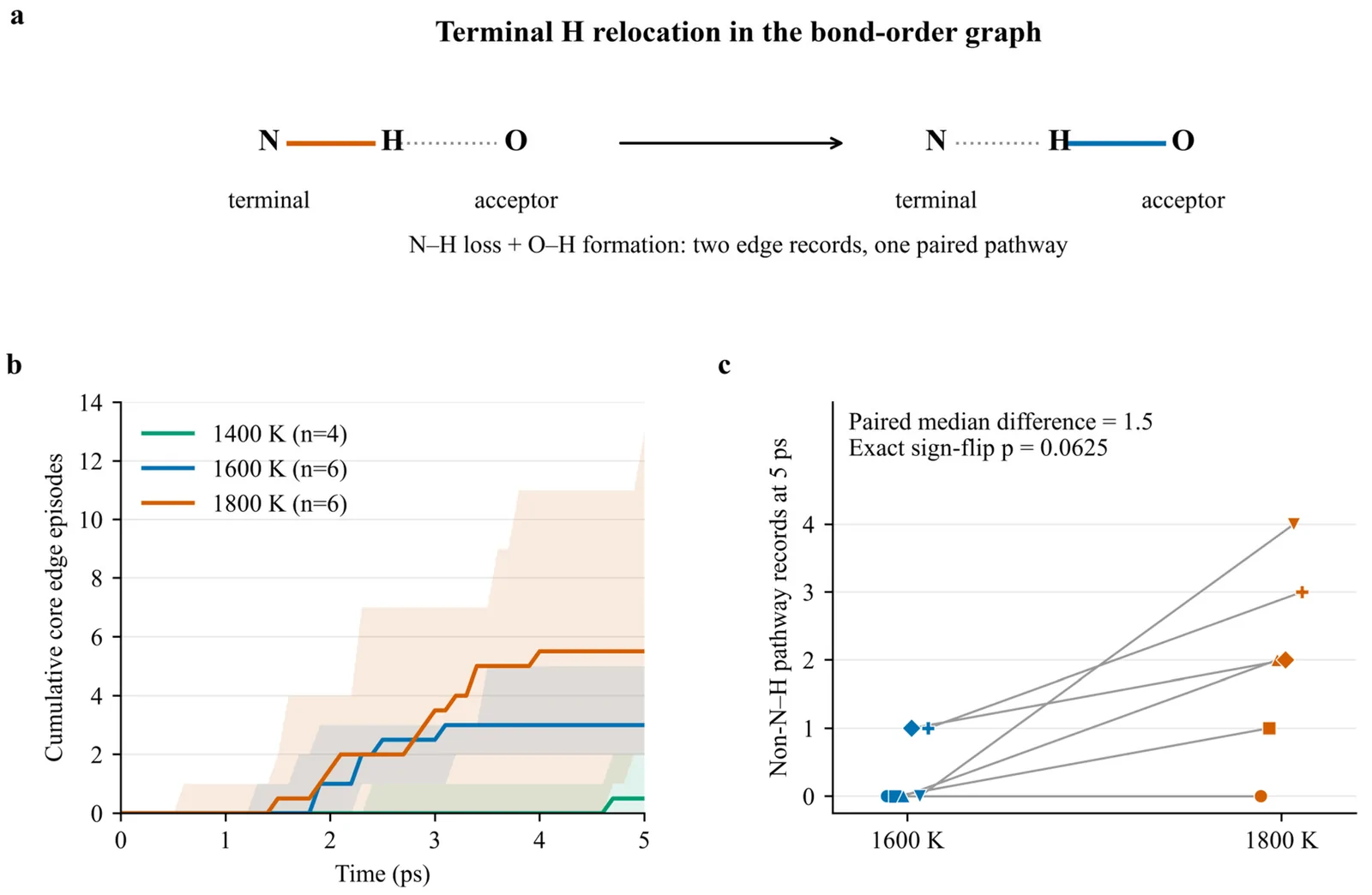
Early thermal connectivity changes. (**a**) Paired N–H loss and O–H formation. (**b**) Cumulative core edge episodes. (**c**) Paired non-N–H endpoint records at 5 ps.

The first 5 ps resolved sparse connectivity changes while retaining more than 99.86% of the eligible core reference graph at every endpoint. Figure 4a illustrates a terminal N–H loss paired with O–H formation; solid connectors denote present graph edges, and dotted connectors denote absent ones. The pair contributes two edge records and one grouped pathway under the shared-H rule. Cumulative edge-episode medians reached 0.5 at 1400 K, 3.0 at 1600 K, and 5.5 at 1800 K (Figure 4b). Solid curves show filmwise medians and shading the observed range, using four films at 1400 K and six at each higher target. The paired cumulative contrast was +3 episodes (exact *p* = 0.1875). Figure 4c separately compares non-N–H grouped endpoint records for the six matched films; connecting lines preserve film identity, and horizontal offsets separate coincident values.

**Figure 5:**
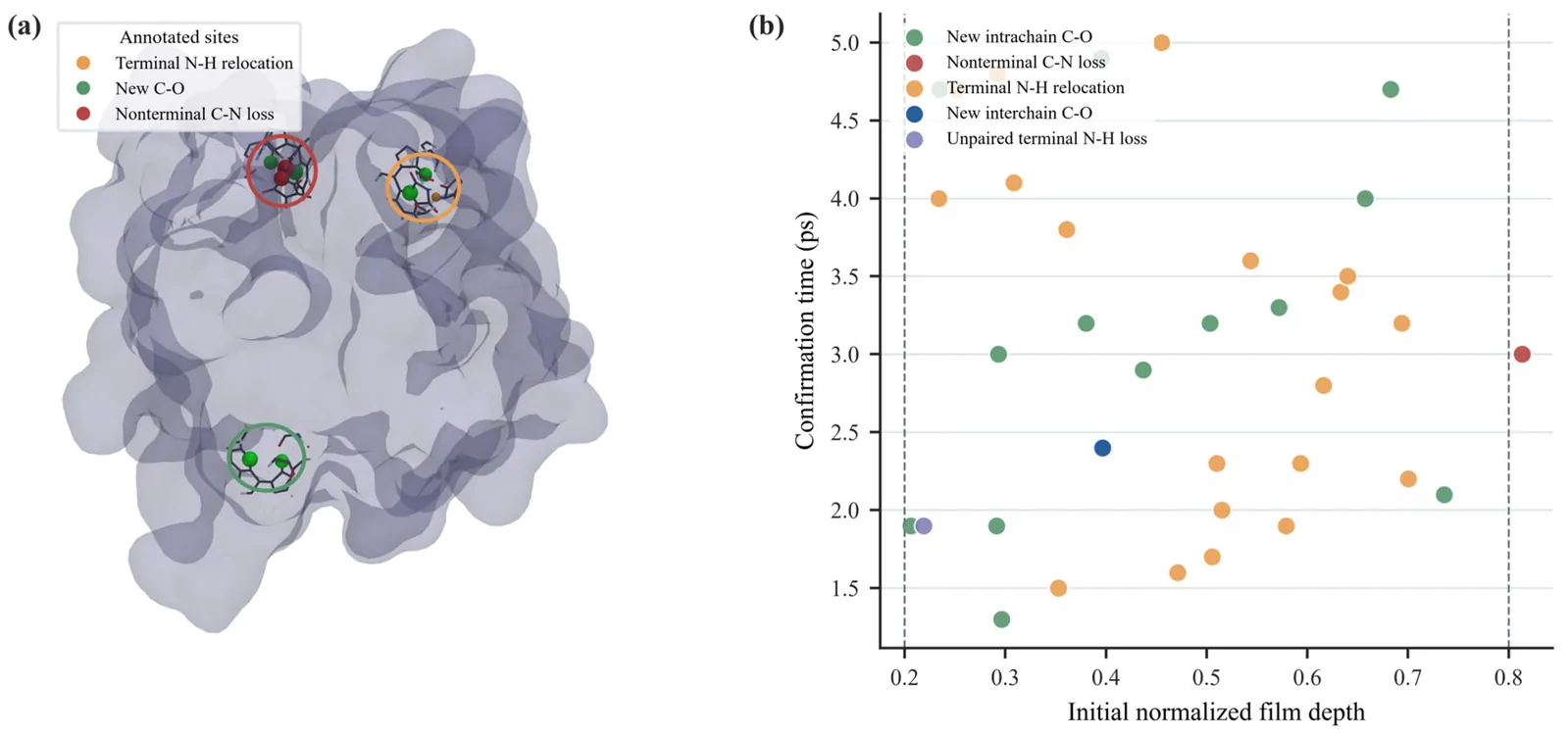
Chain-end identity and spatial distribution of persistent records. (**a**) Annotated film environment. (**b**) Confirmation depth and time.

Terminal imide N–H was the dominant site of early reference-bond loss. All 20 persistent H–N losses mapped to this terminal site, and 19 were paired with O–H formation. The terminal channel accounted for 20 of 21 lost reference bonds and 20 of 35 grouped pathway records across the sixteen 5 ps branches. Figure 5a locates representative records within the film, and Figure 5b resolves their confirmation depth and time. Dashed lines at normalized depths of 0.2 and 0.8 delimit the operational surface regions. Chemical-site mapping thus separates the dominant end-group process from the smaller nonterminal inventory.

The quantitative contribution of the N–H site gives the termination a clear role in interpreting short-chain simulations. One such site occurs among four nitrogen atoms in each 100-atom molecule, yielding 48 terminal N–H sites per film. This high end-group concentration focuses the early loss inventory on the retained termination. Site-resolved accounting makes that contribution explicit and allows the remaining backbone-associated changes to be examined separately.

The local-void comparison further distinguished chemical identity from geometric exposure. Eighteen of the 21 lost-edge sites had the same accessible fraction as their matched retained controls, and three had higher values. Combined with the terminal-site attribution, this result identifies local chemical identity as a necessary variable when comparing bonds at similar void exposure.

Nonterminal changes showed a positive paired temperature contrast in the six-film dataset. Five films had more non-N–H grouped records at 1800 K than at 1600 K, while one was unchanged (Figure 4c). The median paired difference was 1.5 records (two-sided exact *p* = 0.0625); leave-one-film-out medians remained between 1 and 2 records, with *p* values of 0.0625–0.125. The contrast was preserved at formation thresholds of 0.40 and 0.60 and with a two-frame persistence rule; the five-frame rule gave *p* = 0.125. These descriptive comparisons characterize the direction and event-definition sensitivity of the six observed film pairs (Table S4).

Longer observation revealed a growing contribution from nonterminal rearrangements. Between 5 and 10 ps, non-N–H grouped endpoint records increased from 2 to 5 at 1600 K and from 12 to 19 at 1800 K, with all six film pairs within the hot-film coherence criteria. The 20 ps trajectories therefore resolve temporal development beyond the early terminal-dominated inventory. Because endpoint counts track the connectivity present at each reporting time, individual counts can decrease when a previously changed edge recovers. Figure S1a,b show these film-resolved trajectories.

Mass escape became important later in the trajectories. Film 6 crossed the escaped-mass-fraction threshold of 0.10 at 13.7 ps under the 1800 K bath and at 18.6 ps under the 1600 K bath; the maximum fractions were 0.12396 and 0.10751, respectively (Figure S1c,d). The other ten trajectories remained within the coherence criteria through 20 ps. The affected film’s 15 ps endpoint at 1800 K and both 20 ps endpoints are labeled post-coherence-loss diagnostics. This temporal separation between early bond rearrangement and later mass escape supports evaluating chemical connectivity and film integrity as complementary observables.

Across the full six-film inventories at 20 ps, including the flagged post-coherence-loss endpoints, grouped records totaled 51 at 1600 K and 88 at 1800 K, of which 12 and 49 were non-N–H records. Terminal N–H accounted for 78 of 101 reference-bond losses at these endpoints. The combined time series shows the persistent involvement of the terminal channel alongside an increasing diversity of nonterminal changes. Tables S7–S10 retain every film and distinguish endpoint chemistry by structural status.

### 3.3. Carbonyl Connectivity and Anisotropic Electronic Response

**Figure 6:**
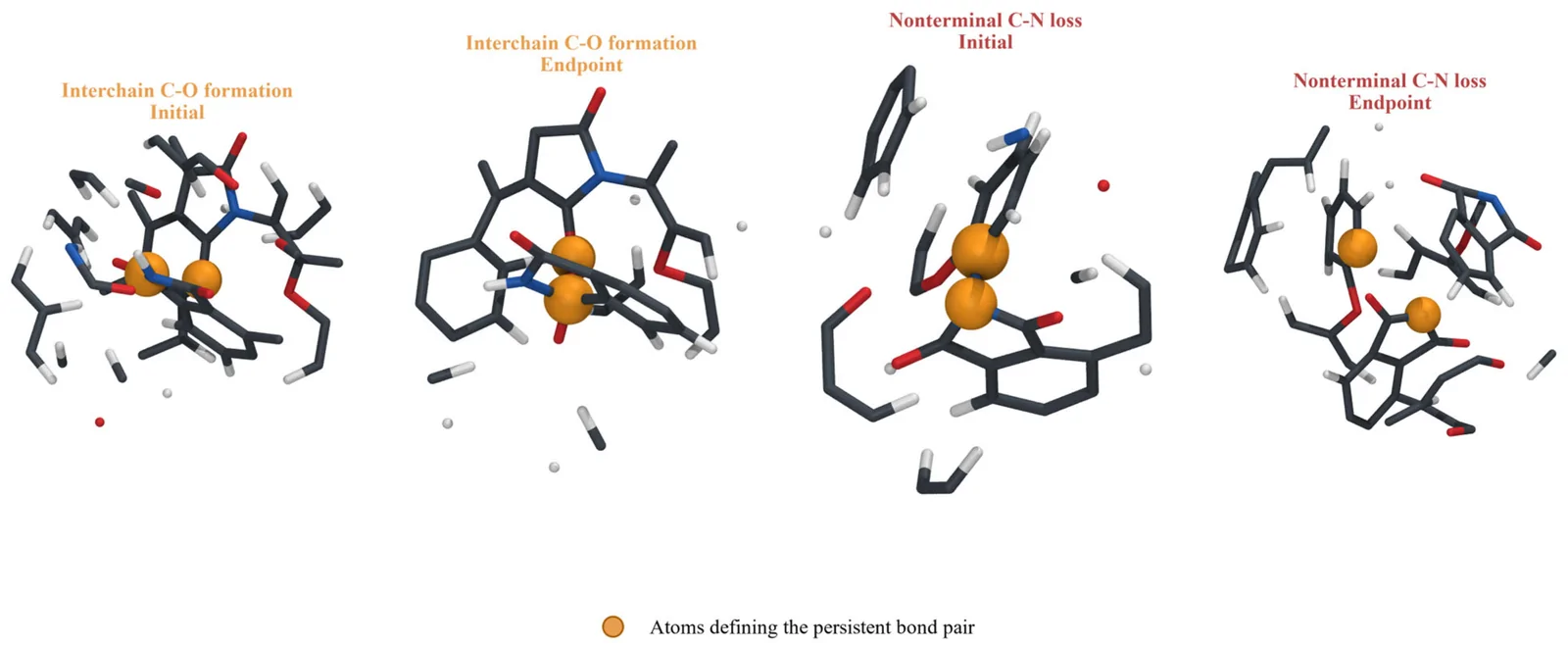
Local environments of nonterminal connectivity changes. Initial and 5 ps configurations for interchain C–O formation and C–N loss.

The nonterminal inventory at 5 ps was dominated by carbonyl-centered C–O formation: 14 new C–O edges and one C–N loss. Thirteen C–O formations were intrachain and one was interchain. Eleven intrachain edges joined an imide carbonyl O to a biphenyl aromatic C, and two joined an imide carbonyl O to an ether-linked aromatic C; the interchain edge linked an imide carbonyl O and an imide carbonyl C. Six edges lay 2–4 bonds from terminal imide N, and eight were farther away. Figure 6 places the interchain C–O formation and the C–N loss in their initial and 5 ps molecular environments. Orange spheres identify the atom pairs used for connectivity analysis.

These early records resolve local carbonyl-associated rearrangement while the surrounding reference network remains largely retained. Longer high-temperature polyimide studies describe ether cleavage and volatile-product formation [16,17]; the present 5–20 ps analysis instead resolves the early spatial and temporal distribution of connectivity changes. Separating the terminal channel exposes this less abundant carbonyl-centered chemistry and makes comparisons across observation times more informative.

**Figure 7:**
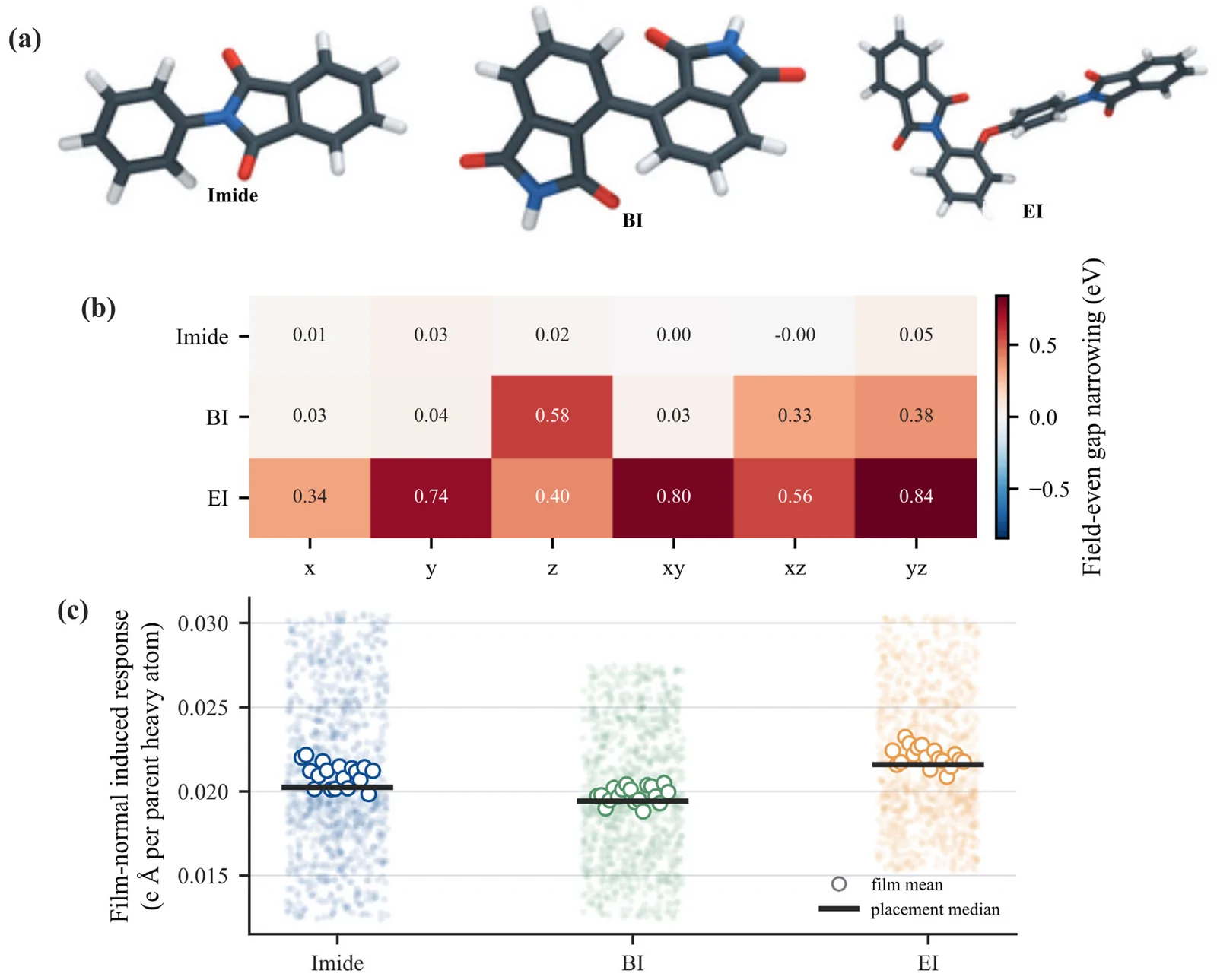
Directional electronic response of isolated fragments. (**a**) Imide, BI, and EI structures. (**b**) Field-even frontier-gap narrowing at ±2 V nm^−1^. (**c**) Film-normal induced-response projections.

The electronic calculations resolve a different distinction: fragment size affects the induced-response amplitude, whereas molecular direction discriminates orbital-gap sensitivity. Figure 7a defines the imide, biphenyl bis-imide (BI), and ether-linked bis-imide (EI) fragments. Their signed induced-response matrices were nearly symmetric, with a maximum antisymmetric residual of 9.5 × 10^−4^ e Å. Principal response ranges normalized by parent-heavy-atom count overlapped at 2 V nm^−1^: 0.0123–0.0307 e Å for imide, 0.0124–0.0275 for BI, and 0.0153–0.0304 for EI. Normalization therefore removes much of the raw size contrast and makes the direction-resolved gap response especially useful for comparing these motifs.

Frontier-gap narrowing clearly separated the selected fragment geometries (Figure 7b). Across six axial and off-axis directions, field-even narrowing was at most 0.049 eV for imide. BI was selectively responsive along z, reaching 0.582 eV, compared with ≤0.379 eV in the other sampled directions. EI showed broader directional sensitivity, with a narrowing of 0.342–0.841 eV and the largest responses along the xy and yz bisectors. EI also had the smallest zero-field gap. Thus, similar size-normalized induced-response ranges coexist with distinct direction-dependent orbital responses.

The frontier gap is an occupied–unoccupied orbital-energy difference at fixed nuclear coordinates. Its field-induced change measures the electronic sensitivity of the isolated, hydrogen-capped fragment to field direction. It is a molecular screening descriptor, rather than a film dielectric-breakdown threshold. Connecting this descriptor to film-scale performance requires condensed-phase screening, intermolecular coupling, charge transport, and structural relaxation. Here, it identifies directions and molecular motifs for targeted electronic comparisons.

Projection onto the 300 K films connected the fragment responses to the distribution of molecular orientations (Figure 7c). Broad placement-level distributions narrowed on averaging within each film. Median projected responses were 0.0194, 0.0202, and 0.0216 e Å per parent heavy atom for BI, imide, and EI, respectively. Light points show individual placements, open circles film means, and horizontal black lines placement medians. Median Kabsch residuals were 1.02, 0.95, and 2.17 Å, respectively, with EI showing the largest conformational deviation from its rigid reference. These projections provide a common orientation-resolved basis for selecting configurations for subsequent condensed-phase electronic calculations.

The charge-model benchmark translates the electronic reference into concrete calibration targets. QEq reproduced the signed induced-dipole direction in all nine axial comparisons, with a median model-to-DFT magnitude ratio of 1.57. QTPIE also preserved all signs, with a median ratio of 0.146. ACKS2 reproduced five of nine directions and gave negligible response amplitude. Agreement in direction and agreement in magnitude are therefore separate calibration requirements. Together with the atomwise charge-ordering results in Supplementary Section S3, this benchmark supplies a quantitative test for selecting charge treatments in future field-on molecular dynamics.

### 3.4. Implications for Molecular Screening and Nanofilm Design

Chain termination and observation time emerge as linked variables for thermal comparisons. The 5 ps bond-loss inventory identifies the terminal imide N–H population as the dominant early channel, while the 10–20 ps inventories resolve additional nonterminal rearrangement and the onset of mass escape. The one-per-molecule N–H population makes the present system an end-rich model. Comparisons with longer-chain polyimides should therefore control end-group concentration together with packing and chain entanglement. Matched end-capping and chain-length studies can use the site-resolved counts reported here to test whether suppressing the terminal channel changes the remaining carbonyl-centered chemistry.

Segment orientation is a complementary screening variable for electronic response. Polyimide dielectric behavior depends on polarizable groups and orientation [5,6,12]. The contrast between overlapping normalized induced responses and distinct gap-narrowing patterns shows why orientation-resolved descriptors add information beyond fragment size. In particular, the selective BI response and broad EI response identify different angular sensitivities for testing through conformational sampling and processing-controlled orientation studies.

Packing descriptors make these chemical and electronic comparisons more discriminating. The fivefold range of accessible fraction and the variation in connected span show that matching density or free-volume fraction alone can leave substantial geometric differences between nanofilms. Including void connectivity alongside chain length and end-group population offers a more structured basis for comparisons across polymer architectures and preparation histories. These geometric descriptors complement free-volume measures used in published molecular and dielectric-property studies [7,12].

The resulting research strategy is to compare chemical modifications within morphology-matched ensembles, resolve terminal and nonterminal records over common observation times, and select electronic configurations using directional response. The signed-response benchmark then provides a calibration step before coupling electric fields to reactive trajectories. This combination of site attribution, geometric matching, and directional screening narrows the variables that subsequent simulations and experiments need to test.

## 4. Conclusions

Aromatic imide oligomer nanofilms exhibit distinct structural, chemical, and electronic sensitivities that can be resolved using complementary molecular descriptors. Across eighteen independently initialized films, probe-accessible volume varied fivefold, and connected void span ranged from 4.47 to 12.59 Å. The accessible fraction and connectivity therefore provide complementary measures for selecting and matching nanofilm morphologies.

Chemical-site attribution identified terminal imide N–H as the dominant early reference-bond-loss channel, accounting for 20 of 21 losses across sixteen 5 ps trajectories. Separating this channel revealed 14 C–O formations and one C–N loss. In the six matched film pairs, nonterminal records increased between 5 and 10 ps, and the trajectories to 20 ps resolved further chemical evolution together with mass escape in one film at both bath targets. The end-rich short-chain model thus provides a site-resolved baseline for testing chain termination and observation-time effects in comparisons with longer-chain systems.

Electronic sensitivity depended strongly on field direction even when size-normalized induced responses overlapped. At ±2 V nm^−1^, the isolated EI fragment exhibited a frontier-gap narrowing of 0.342–0.841 eV across six directions, whereas imide narrowing remained ≤0.049 eV, and BI was the most responsive along one axis. These molecular descriptors prioritize motifs and orientations for electronic screening; film-level dielectric performance additionally depends on condensed-phase interactions and transport. The signed-response benchmark provides quantitative targets for calibrating the charge models needed to bridge those scales.

Together, the results establish a practical basis for candidate design comparisons: account explicitly for chain-end chemistry, match both void fraction and connectivity, and evaluate electronic response as a function of molecular direction. Applying these criteria can make future end-group, chain-length, and orientation studies more discriminating by separating chemical effects from packing and fragment-size differences.

## Data Availability

The original contributions presented in this study are included in the article and Supplementary Material. Further inquiries can be directed to the corresponding author.

## Supplementary Materials

The following supporting information can be downloaded at https://www.mdpi.com/article/10.3390/nano16191236/s1, Figure S1. Time-dependent connectivity and film coherence. (a,b) Non-N–H endpoint records at 1600 and 1800 K. (c,d) Escaped mass fractions at 1600 and 1800 K; Table S1. Thermal trajectories and cumulative core edge episodes at 5 ps, Table S2. Morphology descriptors of all eighteen accepted films, Table S3. Grouped endpoint-persistent pathway records across sixteen 5 ps branches, Table S4. Descriptive thermal contrasts in six matched film pairs, Table S5. Fixed-geometry isolated-fragment frontier gaps under signed fields of magnitude 2 V nm^−1^, Table S6. Core and surface temperatures over 4.5–5.0 ps, Table S7. Numerical completion and film-coherence checks through 20 ps, Table S8. Filmwise endpoint inventories at 1600 K, Table S9. Filmwise endpoint inventories at 1800 K, Table S10. Complete-cohort endpoint totals and structural status. The Supplementary Information contains molecular-model and trajectory accounting (S1); morphology, site-resolved chemistry, and statistical analyses (S2); finite-field descriptors and charge-model comparison (S3); and time-resolved thermal results with filmwise structural status (S4, including Figure S1).
