# Supplementary material for: Chain-End Reactivity and Anisotropic Electronic Softening Define Candidate Design Targets in BPDA/4,4′-ODA Polyimide Insulation
Source: Nanomaterials (Basel). 2026 Sep 30;16(19):1236. doi: 10.3390/nano16191236 (PMC13666579; doi:10.3390/nano16191236)
Supplement: Supplementary file 1 [file nanomaterials-16-01236-s001.zip › nanomaterials-4532992-supplementary.pdf]

Supplementary Information

Chain-End Reactivity and Anisotropic Electronic Softening Define Candidate Design Targets in BPDA/4,4′-ODA Polyimide Insulation

Dongqiao Bai [1] and Tianyu Lin [2,*]

[1] Xihang University, Xi'an 710077, China
[2] China State Key Laboratory of Electric Insulation and Power Equipment, Xi'an Jiaotong University, Xi'an 710049, China
* Correspondence: ltyyy0927@mail.xjtu.edu.cn

S1. Molecular Model, Film Cohort, and Simulation Accounting

Mixed-end molecular identity. Each 100-atom $C_{56}H_{30}N_4O_{10}$ molecule contains one unsubstituted terminal imide N–H, with N bonded to two carbonyl carbons and H. This bond was present in the $C_{56}H_{26}N_4O_{10}$ source structure. The capping procedure added four hydrogens to undercoordinated terminal aromatic carbons. Each 4800-atom film therefore contains 48 terminal imide N–H sites and 192 added aromatic C–H caps. The 5328-edge reference graph includes both end classes. This is a short mixed-end oligomeric assembly.

Molecular structures. Main Figure 1a defines the modeled oligomer and Figure 1b shows the standard BPDA/4,4′-ODA polymer reference. Main Section 2.1 describes their connectivity and the model end groups.

Film construction and acceptance. The lateral cell dimensions were 37.0 × 37.0 Å. The simulation box was periodic in x and y and contained the finite film plus repulsive walls along z. Independent packing and velocity initializations were used for every film. Six previously accepted films were retained before extension. A prospective batch of 24 new packings was then processed without replacement. One failed during construction, leaving 23 constructed films. All 23 passed the global structural gate, 13 passed the local gate, and all 13 completed the mandatory 300 K relaxation. One of these 13 failed the final mandatory gate solely because its endpoint mass-centre shift was −0.528 Å, outside the fixed ±0.5 Å bound. The other 12 extension films were combined with the six retained films to give the 18-film morphology ensemble. The reproducibility archive preserves the complete identifier-level accounting.

Independent initialization used separately seeded placements and orientations of the same 48 molecular templates. Orientations were sampled using normalized Gaussian quaternions, and positions were drawn within the construction region. Placements violating the element-pair minimum intermolecular separations were rejected. The resulting packings underwent common compaction and reactive preparation procedures, providing an ensemble for comparing initialization-dependent morphology at fixed molecular composition.

The element-pair minimum intermolecular separations (Å) were C–C 2.58, C–H 1.96, C–O 2.58, C–N 2.70, H–H 1.60, H–O 1.87, H–N 1.94, O–O 2.75, O–N 2.72, and N–N 2.72. These thresholds governed placement rejection. Molecular rotations and candidate positions were resampled on rejection.

The mapping from film number to construction seed is 1: 93001, 2: 93002, 3: 93004, 4: 93006, 5: 93009, 6: 93012, 7: 94003, 8: 94004, 9: 94006, 10: 94007, 11: 94010, 12: 94011, 13: 94013, 14: 94014, 15: 94015, 16: 94016, 17: 94018, 18: 94024. Films 1–6 form the matched

1600/1800 K thermal cohort, and films 1–4 completed the 1400 K protocol. All eighteen films contribute to the morphology ensemble.

The 300 K acceptance criteria were 1st–99th percentile thickness ratio ≤1.15, escaped mass fraction ≤0.05, maximum internal z gap ≤1 Å, and absolute mass-center shift ≤0.5 Å, together with charge, component, reference-edge, wall, temperature, density, and slope checks. Separate hot-trajectory criteria allowed thermal expansion: thickness ratio ≤1.20, escaped mass fraction ≤0.10, maximum internal gap ≤2 Å, and endpoint mass-center shift ≤2 Å. These bounds were fixed before the thermal runs.

Reactive dynamics. LAMMPS 22 Jul 2025, Update 4, used units real, atom_style full, boundary p p p, a 0.1 fs timestep, and CHON-2019 ReaxFF. The force-field SHA-256 was 3f57732a19d1bfcf65f8fe3c3a0383e2b7caf6193857bc7018a45fba822ba23f; the QTPIE Gaussian-exponent file SHA-256 was af5a621c43a9c5b129eda4fc2f80cbe84b85cffe5a902756110017ccdb14f622. Repulsive *z* walls used ε = 1 kcal mol^{-1}, σ = 3 Å, and a 6 Å cutoff. QTPIE was updated each step over 0–10 Å with a 10^{-6} tolerance and 200 maximum iterations. Neighbor lists were checked every step.

The first 5000 steps (0.5 ps) used whole-system NVT from 300 K to the nominal target, with temperature/centre-of-mass bias removal, a 10 fs damping time, and three thermostat chains with five iterations. For the next 45,000 steps (4.5 ps), all atoms were integrated in NVE. Fixed-membership upper and lower sink groups, each 13.5% of the total mass, were maintained at the target by Langevin thermostats with 10 fs damping and zeroed random force. Coordinates and bond orders were written every 1000 steps (0.1 ps).

Only the surface groups were directly thermostatted after the ramp. Core temperatures therefore reflect finite-time energy transfer from the surfaces. Means over 4.5–5.0 ps were calculated from 21 outputs per branch and summarized as ranges across films. Table S6 reports the core and surface values separately.

The planned thermal matrix contained 54 branches: eighteen films at three bath targets. Sixteen branches completed 5 ps, comprising four films at 1400 K and six matched films at 1600 and 1800 K. Four interrupted branches lacked the scheduled endpoint and were excluded on completion grounds; 34 branches were not started. The campaign was stopped after some outcomes were available, so the completed subset is not a random sample of the planned matrix. Temperature contrasts are consequently reported as descriptive six-film comparisons. The hot-film coherence criteria were thickness ratio ≤1.20, escaped mass fraction ≤0.10, maximum internal z gap ≤2 Å, and absolute mass-center displacement ≤2 Å, alongside numerical and archive-integrity checks. These hot-state bounds are distinct from the 300 K construction criteria above.

Table S1. Thermal trajectories and cumulative core edge episodes at 5 ps.

Nominal target (K)	Complete films	Final 0.5 ps mean core-temperature range (K)	Films with events	Median cumulative qualifying core edge episodes (range)	Median network retention (%)
1400	4	1349–1372	2	0.5 (0–2)	100.0000
1600	6	1536–1575	6	3.0 (2–5)	99.9736
1800	6	1725–1775	6	5.5 (2–13)	99.9473

S2. Film Structure, End-Aware Thermal Chemistry, and Robustness

Morphology analysis. Accessible volume used a cell-centered grid between mass-weighted $z05$ and $z95$ with maximum spacing 0.75 Å. Van der Waals radii were C 1.70, H 1.20, O 1.52, and N 1.55 Å; the probe radius was 1.20 Å. The grid origin was the lower box bound plus half one grid spacing laterally and $z05$ plus half one spacing normally. Component labeling used six-neighbor adjacency, periodic in x and y and nonperiodic in z. Surface grids were no coarser than 2.0 Å and used a periodic 3 × 3 box average before RMS roughness was calculated.

Table S2. Morphology descriptors of all eighteen accepted films.

Descriptor	Median	Interquartile range	Minimum	Maximum
Effective density (g cm^{-3})	1.2236	1.2019–1.2563	1.1546	1.3244
Probe-accessible fraction	0.01781	0.01496–0.02223	0.00533	0.02890
Largest component fraction	0.3024	0.2503–0.3914	0.1826	0.6688
Maximum film-normal span (Å)	7.796	6.110–9.380	4.467	12.587
Mean two-interface RMS roughness (Å)	2.713	2.465–2.849	1.964	3.468

Three complementary counts were evaluated. Cumulative qualifying edge episodes count persistent reference-bond losses and new-bond formations as they arise, including episodes that recover before the endpoint. Endpoint-persistent edge records count the qualifying changes still present at the stated reporting time. Grouped pathway records combine loss and formation events involving the same H atom when their confirmation times differ by at most 0.3 ps. One paired H relocation therefore contributes two edge records and one grouped pathway record.

Table S3. Grouped endpoint-persistent pathway records across sixteen 5 ps branches.

Record class	1400 K	1600 K	1800 K	Total
Paired terminal imide N–H→O–H relocation	1	7	11	19
Unpaired terminal imide N–H loss	0	1	0	1
Intrachain new C–O connectivity	0	2	11	13
Interchain new C–O connectivity	1	0	0	1
Nonterminal C–N loss	0	0	1	1
Total	2	10	23	35

All 20 H–N losses mapped exactly to the single terminal imide N–H in their parent molecule. Nineteen were paired relocations and one was unpaired. Sixteen unique terminal sites reacted across all branches. No reference C–O edge remained lost at an endpoint. Of 14 new C–O records, six were 2–4 graph bonds from terminal imide N and eight were farther than four bonds. None directly involved terminal N, terminal H, or the carbonyl O atoms adjacent to terminal N. The single C–N loss was 7–8 graph bonds from terminal N.

Film-level contrasts and sensitivity. Two-sided exact sign-flip tests enumerated all 2^6 sign assignments for the six paired film differences. Zero differences remained zero under

sign reversal. The reported p values refer to the paired sign-flip null; interpreting them as tests of a population median would additionally require a symmetry assumption.

Table S4. Descriptive thermal contrasts in six matched film pairs.

Response, 1800–1600 K	Median difference	Positive fraction	Exact two-sided *p*
Cumulative qualifying core edge episodes	+3.0	0.667	0.1875
Persistent reference-edge losses	+1.0	0.667	0.5313
Non-N–H grouped endpoint-persistent records	+1.5	0.833	0.0625

The individual non-N–H counts were 0, 0, 0, 1, 0, and 1 at 1600 K and 0, 1, 2, 2, 4, and 3 at 1800 K. Their paired differences were 0, 1, 2, 1, 4, and 2. Formation thresholds of 0.40 and 0.60 left this result unchanged. A two-frame persistence definition added one 1600 K terminal N–H record but did not change the non-N–H contrast. Five-frame persistence gave $p = 0.125$ for the non-N–H contrast. Thus dominant end-group loss and the sparse non-N–H connectivity changes were stable to the tested definitions.

Leaving out one film at a time gave non-N–H paired median differences of 1–2 records and two-sided exact sign-flip p values of 0.0625–0.125. This analysis measures sensitivity to individual films. The independent sample remains six films across temperatures and reporting times.

The prespecified total-event screen used exact Spearman tests over all 6! film-label permutations for accessible fraction, maximum vertical span, roughness, and density at each temperature. The smallest exact p value was 0.1361 for roughness versus 1800 K total burden ($\varrho = -0.714$). The post hoc end-group-excluded screen gave $\varrho = -0.870$ for maximum vertical span versus 1800 K non-N–H burden, with unadjusted exact p = 0.0333 and leave-one-film-out ϱ from −0.872 to −0.800. Benjamini–Hochberg adjustment across eight tests gave q = 0.267. These exploratory associations are reported separately from the geometric packing comparisons.

The bond-local accessible-volume comparison contained 21 persistent reference-edge losses: twenty terminal imide N–H losses and one nonterminal C–N loss. Eighteen event/control pairs had equal accessible fractions, three event values were higher, and none were lower. This comparison characterizes geometric exposure primarily for the terminal channel.

S3. Signed Finite-Field Response, Orientation Projection, and Charge-Model Comparison

Electronic-structure protocol. The neutral-singlet imide-containing, biphenyl bis-imide (BI), and ether-linked bis-imide (EI) fragments had 26/17, 30/22, and 51/35 total/parent-heavy atoms, respectively. ORCA 6.1.0 fixed-geometry inputs used r²SCAN-3c, TightSCF, SlowConv, MaxIter 300, a center-of-mass field origin, Hirshfeld populations, and Mayer bond orders. Each signed field had magnitude 0.003889380762 atomic units, corresponding to 2 V nm^{-1} with the conversion 514.220674763 V nm^{-1} per atomic unit. All 21 primary axial jobs and all 18 added off-axis jobs terminated normally. The retained source-data identifiers imide, bpda, and oda_ether correspond to imide, BI, and EI, respectively; these archive identifiers are not additional material assignments.

Table S5. Fixed-geometry isolated-fragment frontier gaps under signed fields of magnitude 2 V nm^{-1}.

Fragment	Direction	Zero-field gap (eV)	Gap at −*E* (eV)	Gap at +*E* (eV)	Field-even narrowing (eV)	Field-odd shift (eV)
Imide	*x*	3.1618	3.3625	2.9387	0.0112	−0.2119
Imide	*y*	3.1618	2.7668	3.4881	0.0344	0.3607
Imide	*z*	3.1618	2.9020	3.3910	0.0153	0.2445
Imide	*xy*	3.1618	3.0495	3.2682	0.0030	0.1094
Imide	*xz*	3.1618	3.1382	3.1859	−0.0003	0.0239
Imide	*yz*	3.1618	2.6908	3.5355	0.0487	0.4224
BI	*x*	3.2513	3.2478	3.2014	0.0267	−0.0232
BI	*y*	3.2513	3.2022	3.2142	0.0431	0.0060
BI	*z*	3.2513	2.6791	2.6597	0.5819	−0.0097
BI	*xy*	3.2513	3.2285	3.2076	0.0333	−0.0104
BI	*xz*	3.2513	2.9647	2.8806	0.3287	−0.0421
BI	*yz*	3.2513	2.8746	2.8709	0.3786	−0.0018
EI	*x*	2.3911	2.3055	1.7934	0.3417	−0.2561
EI	*y*	2.3911	1.5628	1.7471	0.7362	0.0922
EI	*z*	2.3911	2.0898	1.8849	0.4038	−0.1025
EI	*xy*	2.3911	1.6963	1.4864	0.7997	−0.1050
EI	*xz*	2.3911	2.0716	1.5895	0.5605	−0.2411
EI	*yz*	2.3911	1.5456	1.5543	0.8411	0.0043

Field-even frontier-gap narrowing was ≤0.049 eV for imide. BI reached 0.582 eV along z, while EI gave 0.342–0.841 eV across the six sampled directions and had the smallest zero-field gap. These values characterize the selected isolated-fragment geometries.

The frontier gap is an occupied–unoccupied orbital-energy difference at fixed nuclear coordinates. Its field-induced change measures the electronic sensitivity of the isolated, hydrogen-capped fragment to field direction. It is a molecular screening descriptor, rather than a film dielectric-breakdown threshold. Connecting this descriptor to film-scale performance requires condensed-phase screening, intermolecular coupling, charge transport, and structural relaxation. Here, it identifies directions and molecular motifs for targeted electronic comparisons.

The signed induced-response matrices were symmetrized after verifying a maximum antisymmetric residual of 9.5×10^{-4} e Å. Principal ranges normalized by parent-heavy-atom count were 0.0123–0.0307 e Å for imide, 0.0124–0.0275 e Å for BI, and 0.0153–0.0304 e Å for EI. These overlapping ranges contrast with the distinct directional gap responses of the selected fragments.

Rigid-reference film projection. Fragment topologies were mapped to each molecule in all eighteen films. A parent-heavy-atom Kabsch fit supplied the rotation from the reference geometry to each placement. Median film-normal projected responses were 0.0194, 0.0202, and 0.0216 e Å per parent heavy atom for BI, imide, and EI, respectively. Median Kabsch RMSDs were 1.02, 0.95, and 2.17 Å, with the largest conformational de-

viation for EI. These projections quantify the orientational distribution of the reference-fragment responses.

Fluctuating-charge comparison. Classical single-point calculations used boundary f f f, run 0, ±0.2 V Å^{-1} fields, and the same fixed coordinates. Classical induced dipoles include point-charge redistribution only.

For QEq/QTPIE/ACKS2, the atomwise odd-response Spearman correlations were 0.773/0.641/−0.027, the correct dipole-direction fractions were 1.000/1.000/0.556, and the median signed dipole ratios were 1.570/0.146/0.000033. QEq overresponded, QTPIE underresponded, and ACKS2 gave negligible response with four wrong directions. No scheme matched direction, atomwise ordering, and magnitude; field-on reactive film trajectories were therefore excluded.

S4. Time-Resolved Thermal Response from 5 to 20 ps

Time dependence was evaluated for all six matched films at 1600 and 1800 K through a total elapsed time of 20 ps, with scheduled endpoints at 5, 10, 15, and 20 ps. The 5–20 ps interval began from the corresponding 5 ps checkpoints and retained the molecular connectivity, CHON-2019 force field, QTPIE settings, 0.1 fs timestep, repulsive walls, and fixed surface groups. Coordinates and velocities were continuous at the checkpoint interface. The restart files did not preserve the Langevin random-number state, so deterministic thermostat seeds were specified before continuation. These are checkpoint-based stochastic continuations.

The same bond-order, persistence, and shared-H grouping criteria were applied at every reporting time. Persistence was evaluated separately at each endpoint, so an earlier change could disappear from a later inventory if its connectivity recovered. All scheduled trajectories were included. Numerical completion and hot-film coherence were evaluated separately; endpoints following the first coherence-threshold crossing were retained as post-coherence-loss diagnostics in the filmwise tables.

The 5–20 ps continuation protocol was recorded on 23 September 2026 before execution (SHA-256: f84c7fc2ba2ad32705d23684209477b2d395d33af4ded909c74f6d0edfb5cacb). The Langevin seed was 953000 + 100c + T/100, where c is film number 1–6 and T is 1600 or 1800 K. The twelve seeds were 953116, 953118, 953216, 953218, 953316, 953318, 953416, 953418, 953516, 953518, 953616, and 953618.

All twelve trajectories terminated normally at step 200000. Numerical checks covered finite outputs, charge closure ≤10^{-4} e, maximum absolute atomic charge ≤2 e, QTPIE iterations below 200, and archive/frame integrity. Coordinates, velocities, and recorded bond orders agreed at the 5 ps checkpoint interface.

The 5 ps endpoint edge identities and confirmation times were checked against the archived trajectories. Between 5 and 10 ps, non-N–H grouped records increased from 2 to 5 at 1600 K and from 12 to 19 at 1800 K. All six matched films satisfied the hot-film coherence criteria at both endpoints. Endpoint inventories represent the changes present at each reporting time and can decrease when a changed connectivity recovers.

Mass escape became important later in the trajectories. Film 6 crossed the escaped-mass-fraction threshold of 0.10 at 13.7 ps under the 1800 K bath and at 18.6 ps under the 1600 K bath; maximum fractions were 0.12396 and 0.10751, respectively (Figure S1c,d). The other ten trajectories remained within the coherence criteria through 20 ps. The affected film's 15 ps endpoint at 1800 K and both 20 ps endpoints are labeled post-coherence-loss diagnostics. This temporal separation between early bond rearrangement and later mass escape supports evaluating chemical connectivity and film integrity as complementary observables.

Across the full six-film inventories at 20 ps, including the flagged post-coherence-loss endpoints, grouped records totaled 51 at 1600 K and 88 at 1800 K, of which 12 and

49 were non-N–H records. Terminal N–H accounted for 78 of 101 reference-bond losses at these endpoints. The combined time series shows persistent involvement of the terminal channel alongside an increasing diversity of nonterminal changes. Tables S7–S10 retain every film and distinguish endpoint chemistry by structural status.

Table S6. Core and surface temperatures over 4.5–5.0 ps.

Film	Target (K)	Core (K)	Surface (K)
1	1400	1354.5	1407.9
1	1600	1535.5	1607.2
1	1800	1739.5	1798.4
2	1400	1363.7	1393.4
2	1600	1556.0	1595.4
2	1800	1765.8	1813.1
3	1400	1371.5	1405.2
3	1600	1571.1	1595.4
3	1800	1775.3	1814.5
4	1400	1349.0	1412.3
4	1600	1568.0	1606.0
4	1800	1752.5	1795.6
5	1600	1574.7	1601.2
5	1800	1750.8	1803.2
6	1600	1547.1	1607.1
6	1800	1724.8	1809.2

S4.1. Numerical Completion and Film Coherence

Table S7. Numerical completion and film-coherence checks through 20 ps.

Film	Seed	Target (K)	Numerical audit	Coherent to 20 ps	First loss (ps)	Maximum escaped mass
1	93001	1600	Pass	Yes	–	0.07142
1	93001	1800	Pass	Yes	–	0.08554
2	93002	1600	Pass	Yes	–	0.08236
2	93002	1800	Pass	Yes	–	0.09441
3	93004	1600	Pass	Yes	–	0.06895
3	93004	1800	Pass	Yes	–	0.08005
4	93006	1600	Pass	Yes	–	0.08045
4	93006	1800	Pass	Yes	–	0.08547
5	93009	1600	Pass	Yes	–	0.05730
5	93009	1800	Pass	Yes	–	0.06219
6	93012	1600	Pass	No	18.6	0.10751
6	93012	1800	Pass	No	13.7	0.12396

All twelve trajectories completed 20 ps. Film 6 crossed only the escaped-mass bound among the reported coherence checks. First-crossing times use the 0.1 ps saved frames. A dash denotes no crossing by 20 ps; "coherent through endpoint" requires no earlier crossing. The table retains both trajectories of every film. Later chemistry for a branch that crossed the bound is classified as post-coherence-loss diagnostic data.

S4.2. Filmwise endpoint inventories

Tables S8–S9 report every scheduled endpoint. E denotes endpoint-persistent edge records (losses plus formations), P denotes grouped pathway records, and non-NH denotes grouped records outside the terminal N–H channel. NH loss/all loss reports terminal N–H and total reference losses. "C" identifies an endpoint within the coherence window and "D" a post-coherence-loss diagnostic endpoint. Table S10 summarizes the complete cohort and retains its structural-status information. Figure S1a,b plots non-N–H records with one line per film and open symbols for post-coherence-loss endpoints. Figure S1c,d plots escaped mass fractions; the horizontal dashed line is the 0.10 bound, and vertical dotted lines mark the first crossing for film 6.

Table S8. Filmwise endpoint inventories at 1600 K.

Film	Time (ps)	E	P	non-NH	NH loss/ all loss	Core-edge retention (%)	Escaped mass	Status
1	5	4	2	0	2/2	99.9474	0.03686	C
1	10	6	3	0	3/3	99.9211	0.05613	C
1	15	12	7	1	6/6	99.8422	0.05826	C
1	20	13	8	2	6/6	99.8422	0.07108	C
2	5	2	1	0	1/1	99.9736	0.04911	C
2	10	5	3	1	2/2	99.9473	0.06039	C
2	15	6	3	0	3/3	99.9209	0.07092	C
2	20	12	7	1	6/6	99.8418	0.07939	C
3	5	3	2	0	2/2	99.9471	0.03890	C
3	10	14	8	1	7/7	99.8150	0.04278	C
3	15	16	9	1	8/8	99.7885	0.06137	C
3	20	19	11	2	9/9	99.7621	0.06337	C
4	5	3	2	1	1/1	99.9736	0.04628	C
4	10	2	1	0	1/1	99.9736	0.05079	C
4	15	5	3	1	2/2	99.9472	0.06686	C
4	20	10	5	0	5/5	99.8681	0.07968	C
5	5	2	1	0	1/1	99.9737	0.03200	C
5	10	6	3	0	3/3	99.9211	0.04060	C
5	15	9	5	1	4/4	99.8948	0.04843	C
5	20	14	8	1	7/7	99.8159	0.05706	C
6	5	3	2	1	1/1	99.9736	0.05413	C
6	10	8	6	3	3/3	99.9208	0.07113	C
6	15	12	9	6	3/6	99.8416	0.09001	C
6	20	17	12	6	6/9	99.7624	0.10474	D

Table S9. Filmwise endpoint inventories at 1800 K.

Film	Time (ps)	E	P	non-NH	NH loss/ all loss	Core-edge retention (%)	Escaped mass	Status
1	5	10	5	0	5/5	99.8685	0.04460	C
1	10	23	14	5	9/11	99.7108	0.05660	C
1	15	26	18	8	10/12	99.6845	0.06868	C
1	20	28	20	10	10/14	99.6319	0.08529	C
2	5	5	3	1	2/2	99.9473	0.04895	C
2	10	10	7	4	3/4	99.8945	0.06657	C
2	15	14	10	6	4/6	99.8418	0.07780	C
2	20	23	16	9	7/11	99.7100	0.09119	C
3	5	2	2	2	0/0	100.0000	0.03454	C
3	10	9	6	3	3/3	99.9207	0.04707	C
3	15	18	14	8	6/9	99.7621	0.06357	C
3	20	26	21	13	8/14	99.6299	0.07453	C
4	5	2	2	2	0/0	100.0000	0.05052	C
4	10	3	2	1	1/2	99.9472	0.06248	C
4	15	6	5	3	2/3	99.9209	0.06716	C
4	20	10	8	5	3/5	99.8681	0.08150	C
5	5	6	5	4	1/2	99.9474	0.03856	C
5	10	10	8	6	2/3	99.9211	0.03992	C
5	15	5	4	3	1/2	99.9474	0.05399	C
5	20	11	9	6	3/5	99.8685	0.06024	C
6	5	9	6	3	3/3	99.9208	0.06913	C
6	10	8	4	0	4/4	99.8944	0.08184	C
6	15	16	10	4	6/7	99.8152	0.10449	D
6	20	21	14	6	8/10	99.7360	0.12271	D

Table S10. Complete-cohort endpoint totals and structural status.

Time (ps)	Target (K)	Coherent/ scheduled	E total	P total	non-NH total	NH loss/ all loss
5	1600	6/6	17	10	2	8/8
5	1800	6/6	34	23	12	11/12
10	1600	6/6	41	24	5	19/19
10	1800	6/6	63	41	19	22/27
15	1600	6/6	60	36	10	26/29
15	1800	5/6	85	61	32	29/39
20	1600	5/6	85	51	12	39/42
20	1800	5/6	119	88	49	39/59

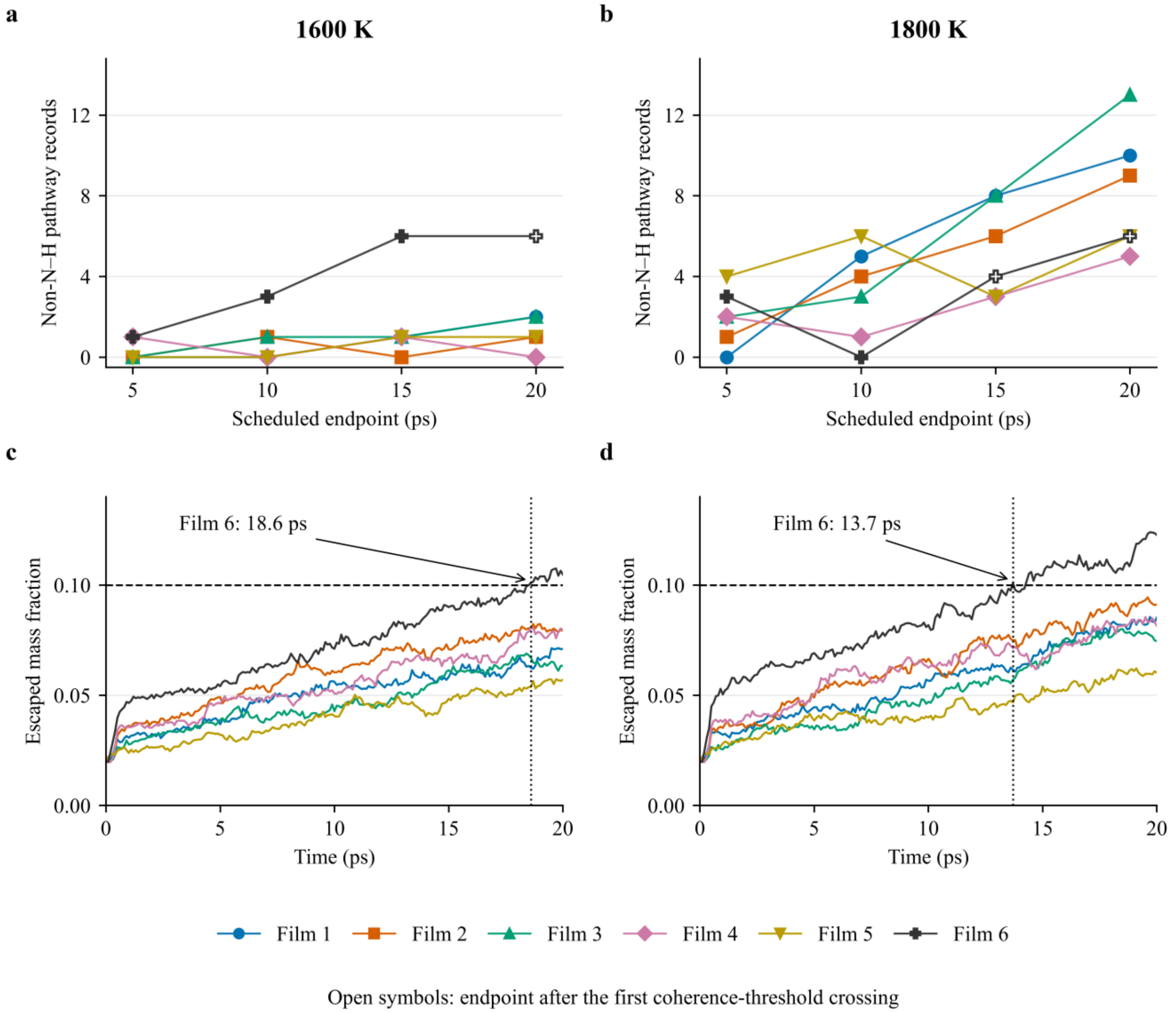

Figure S1. Time-dependent connectivity and film coherence. (a,b) Non-N–H endpoint records at 1600 and 1800 K. (c,d) Escaped mass fractions at 1600 and 1800 K.
